# Supplementary figures and images for: CD56dimCD16dim NK cells are the dominant effector cells against HIV-infected primary T-cells
Source: bioRxiv. 2026 Jul 1:2026.06.26.734820. Preprint. [Version 2] doi: 10.64898/2026.06.26.734820 (PMC13345295; doi:10.64898/2026.06.26.734820)

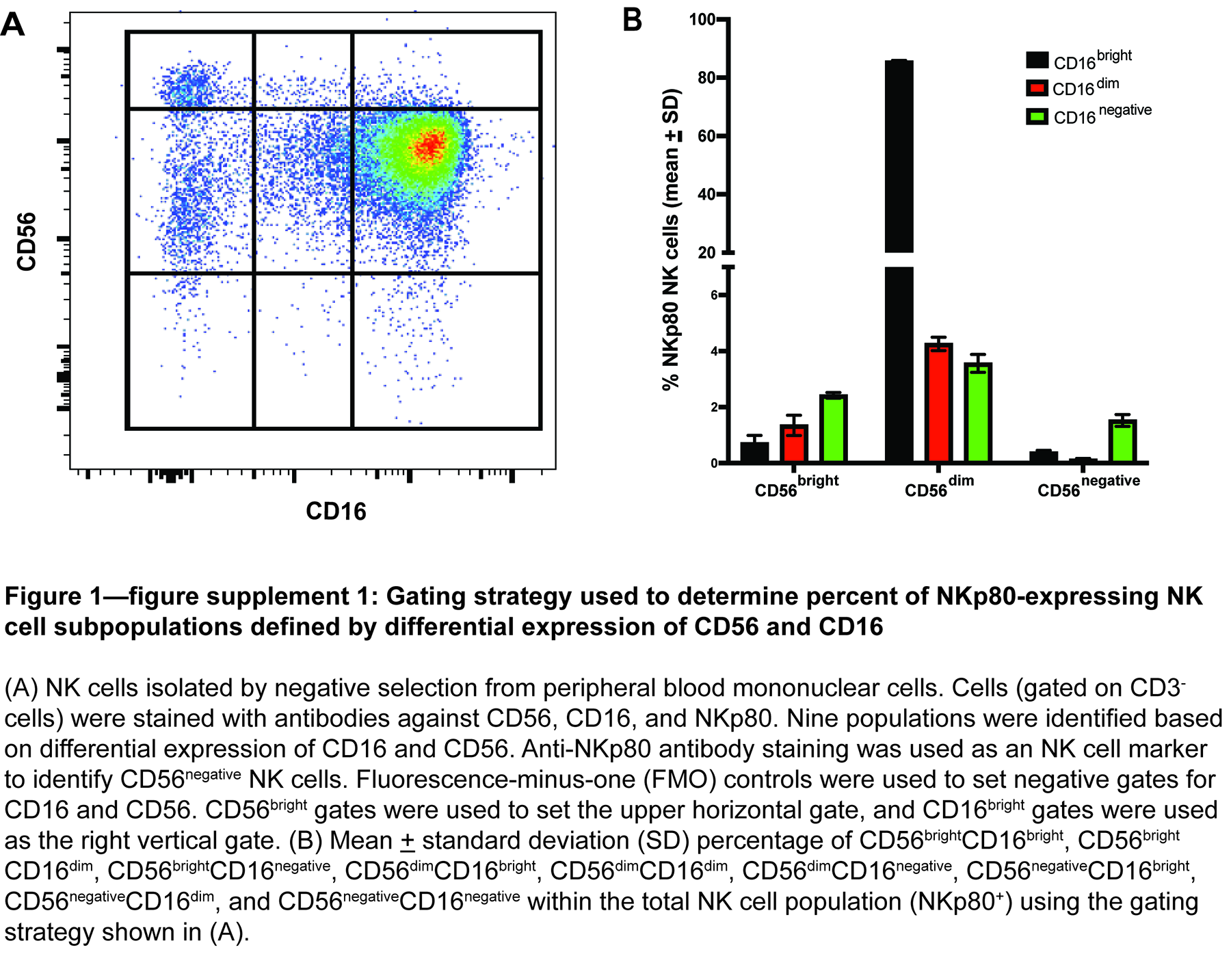

Supplement: Supplement 28 [file media-28.tif]

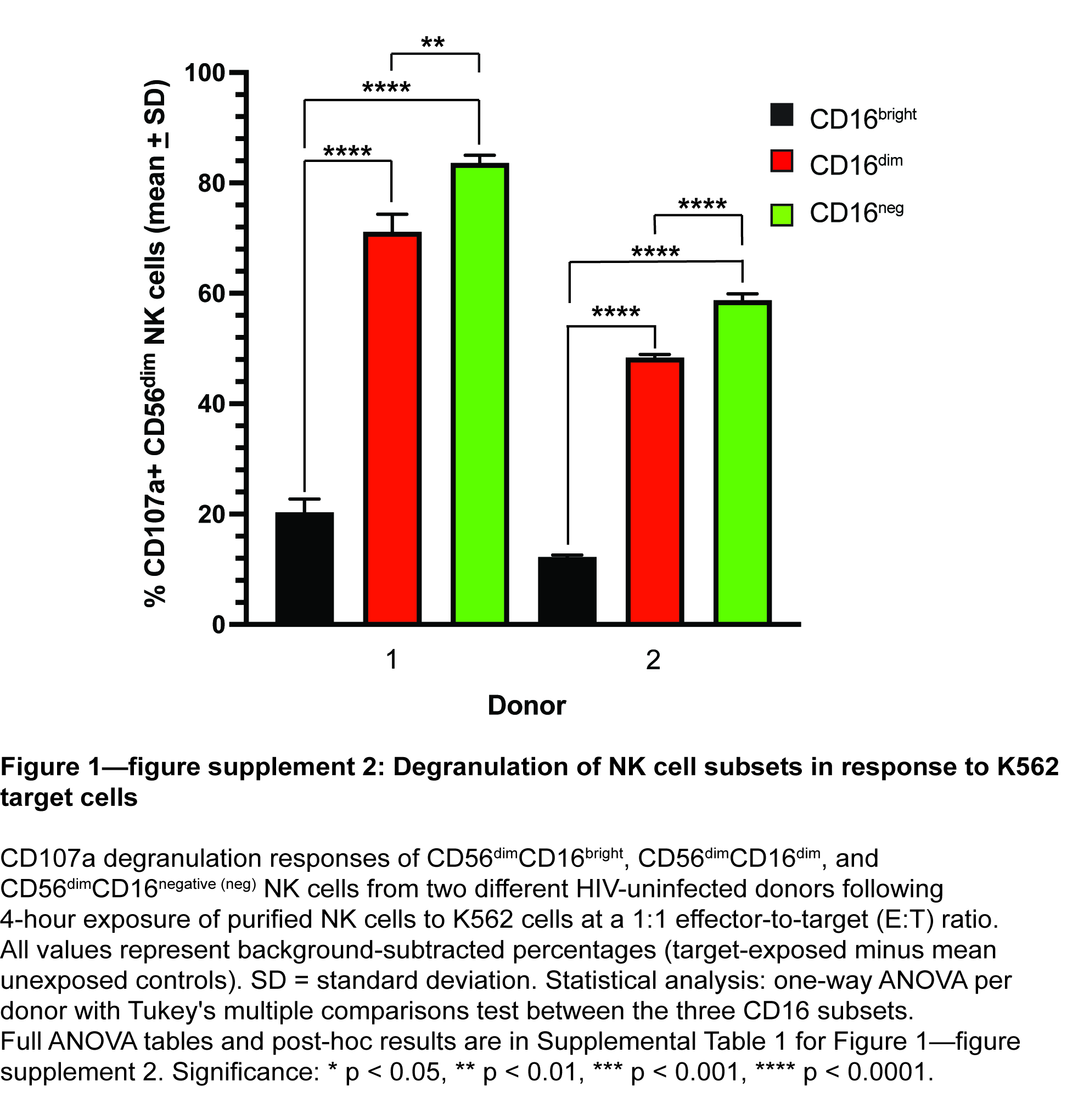

Supplement: Supplement 29 [file media-29.tif]

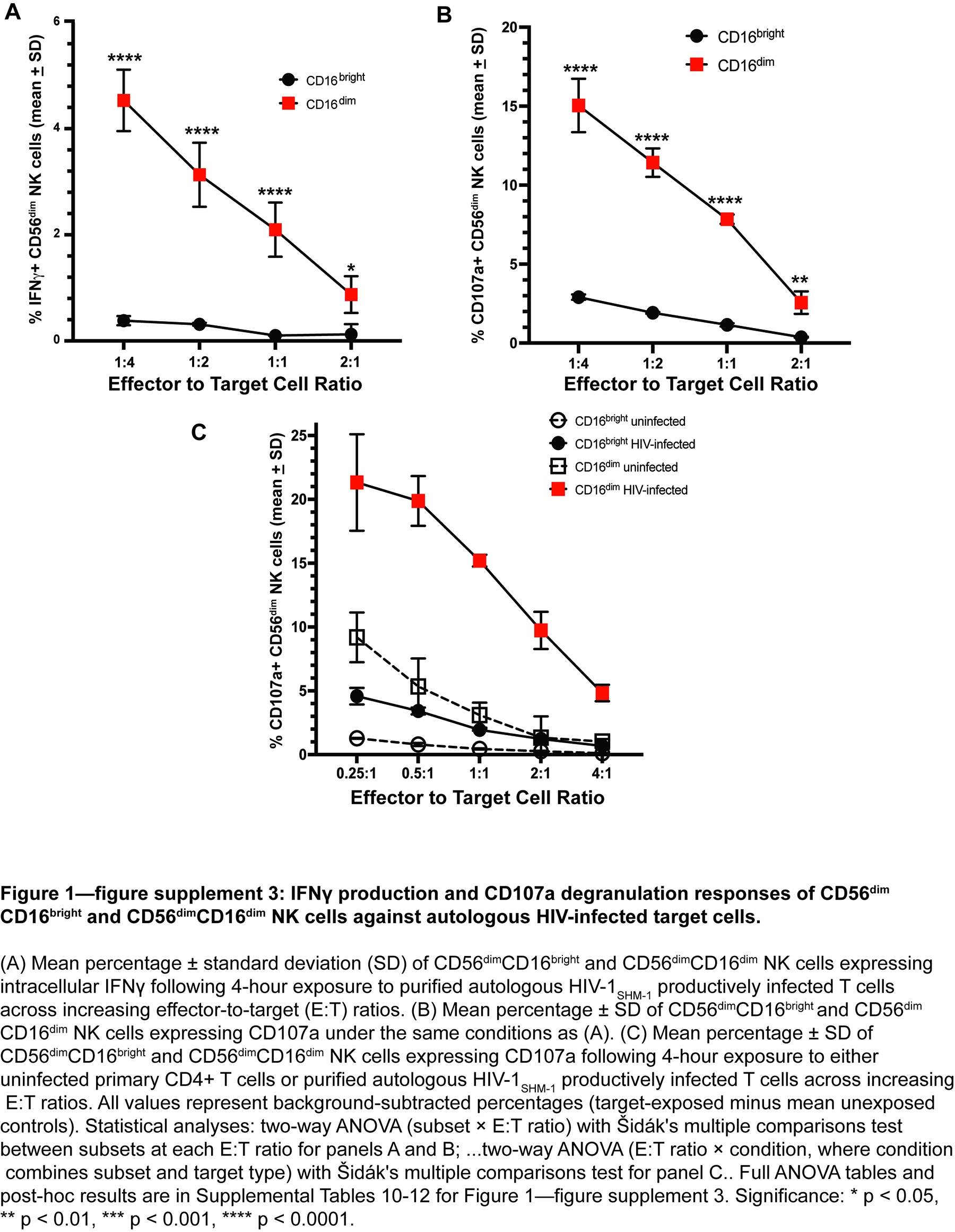

Supplement: Supplement 30 [file media-30.tif]

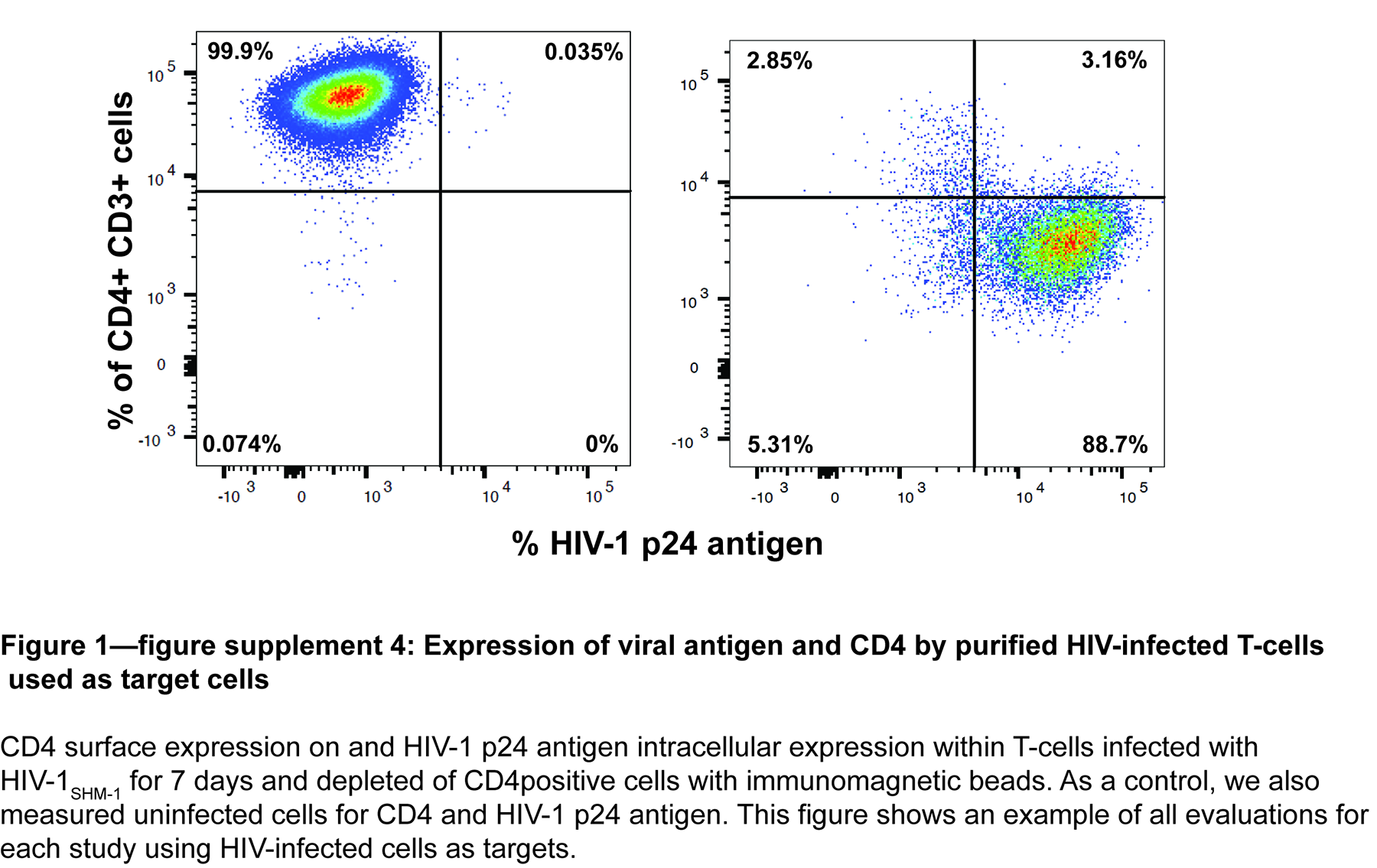

Supplement: Supplement 31 [file media-31.tif]

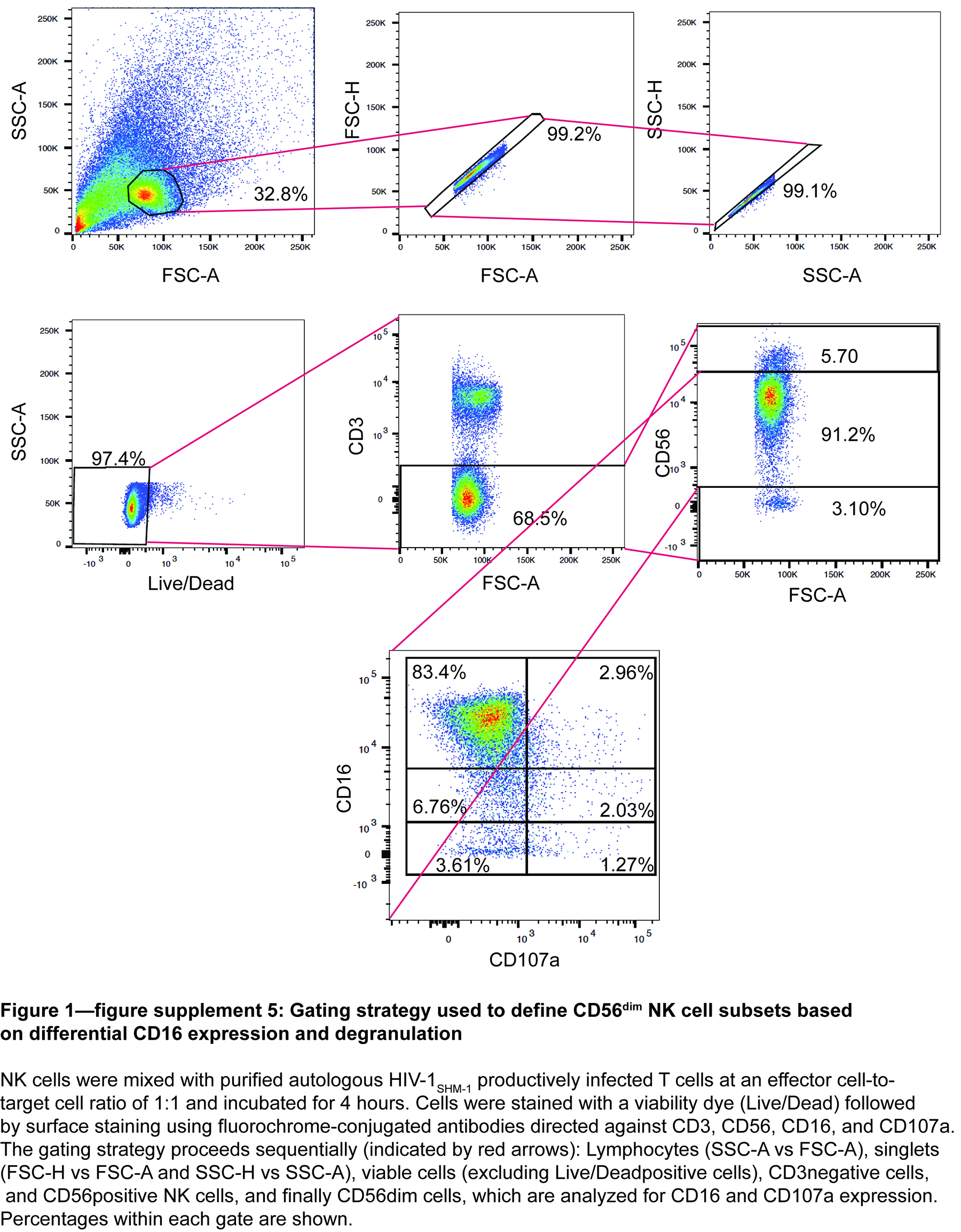

Supplement: Supplement 32 [file media-32.tif]

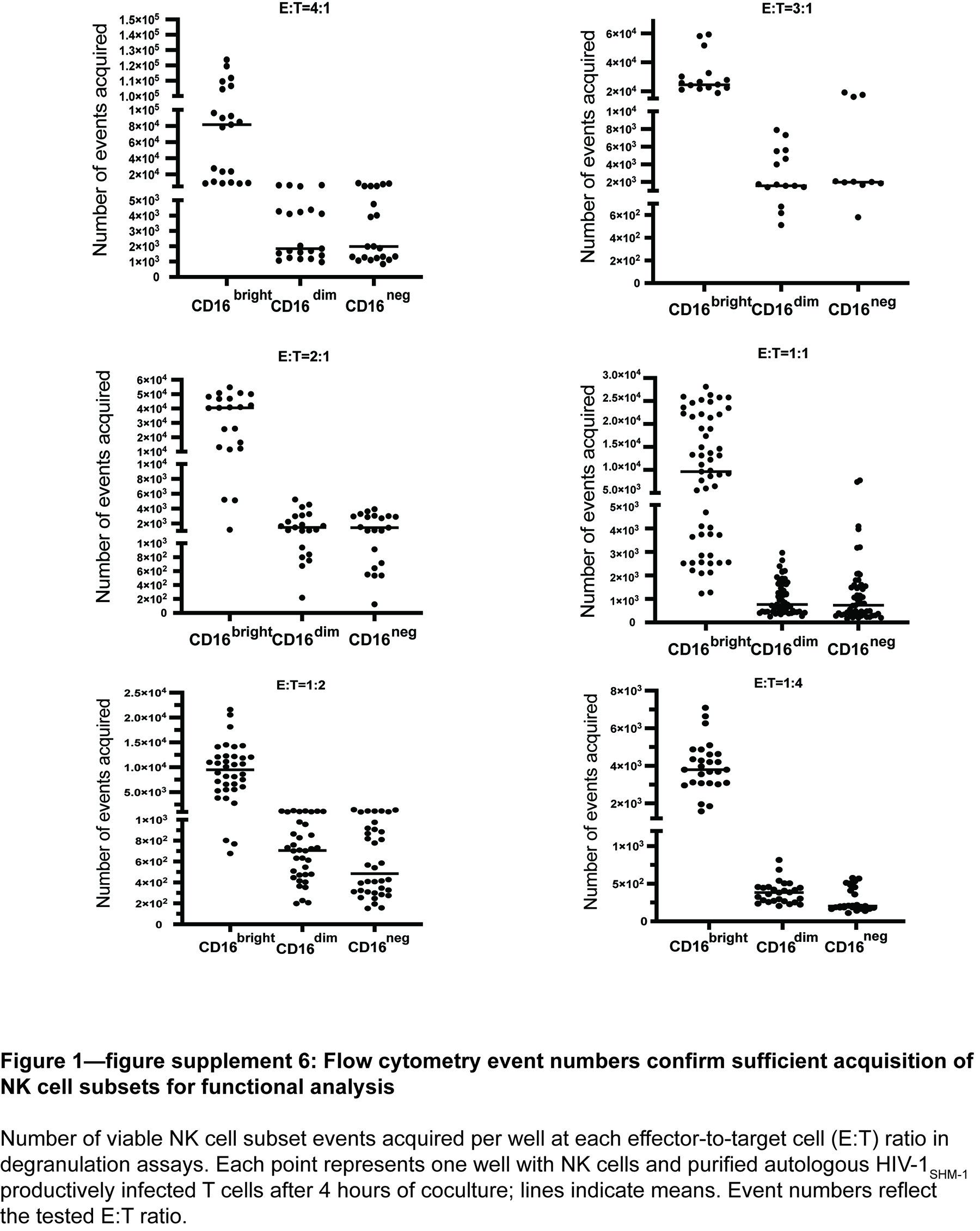

Supplement: Supplement 33 [file media-33.tif]

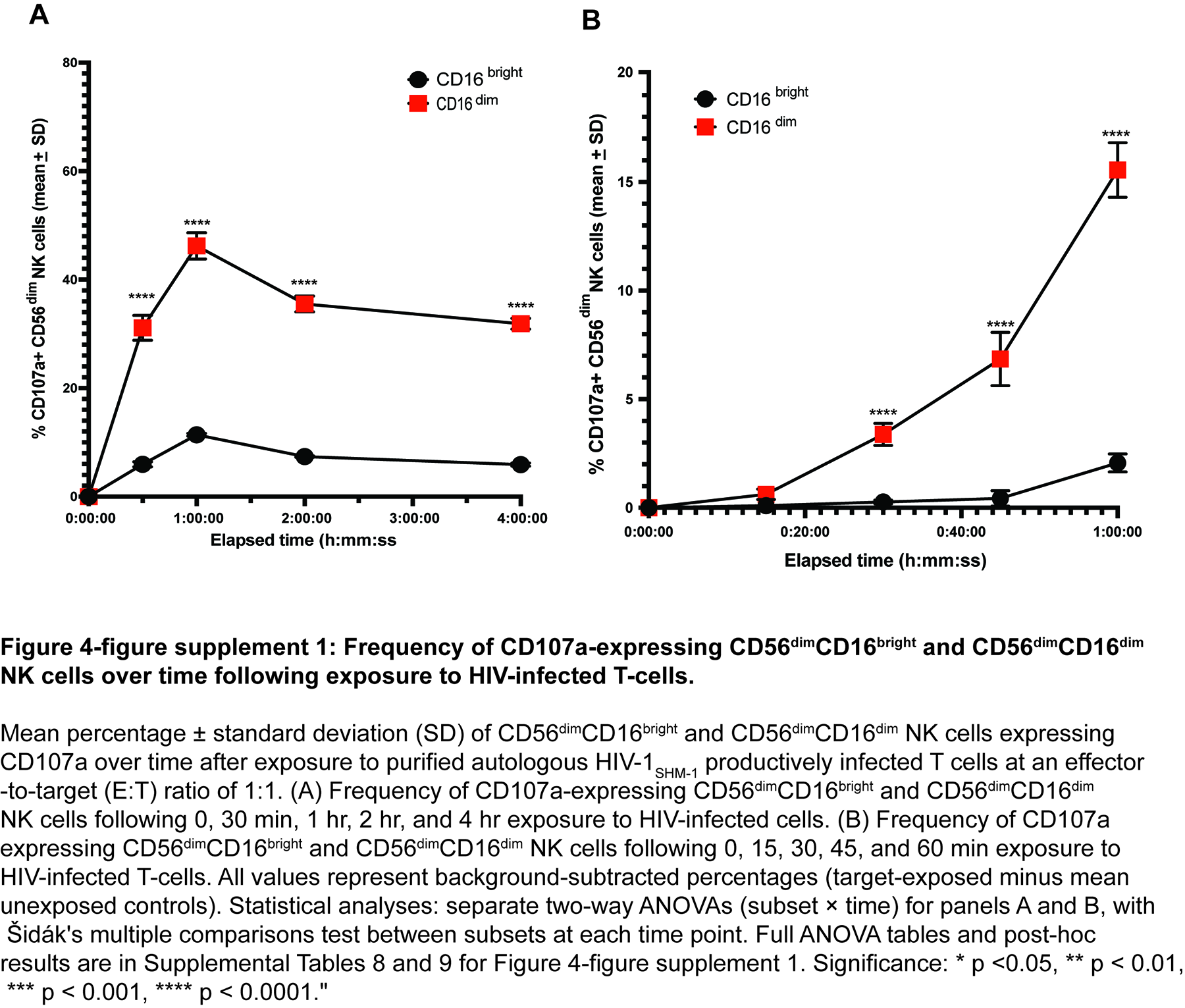

Supplement: Supplement 34 [file media-34.tif]

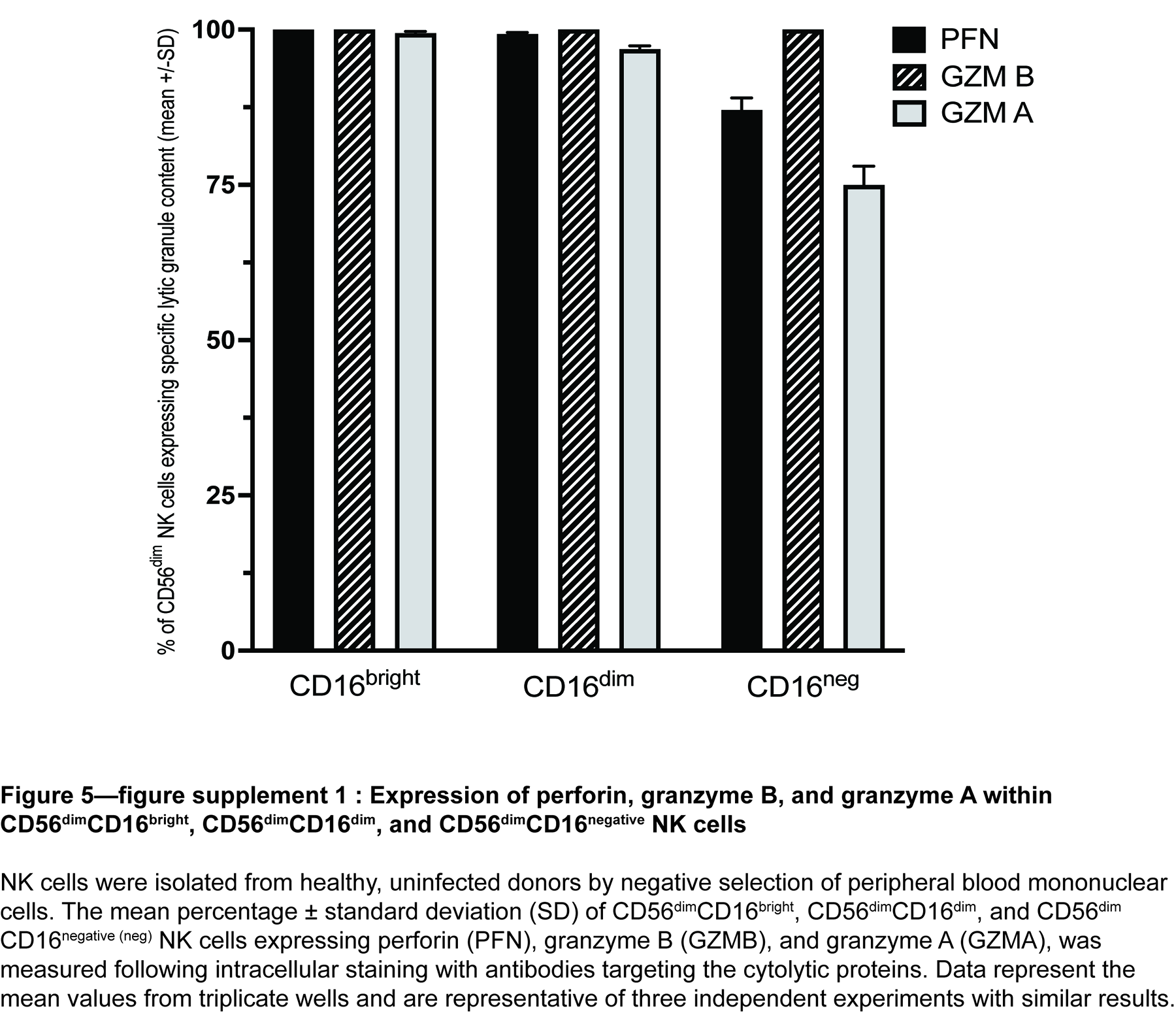

Supplement: Supplement 35 [file media-35.tif]

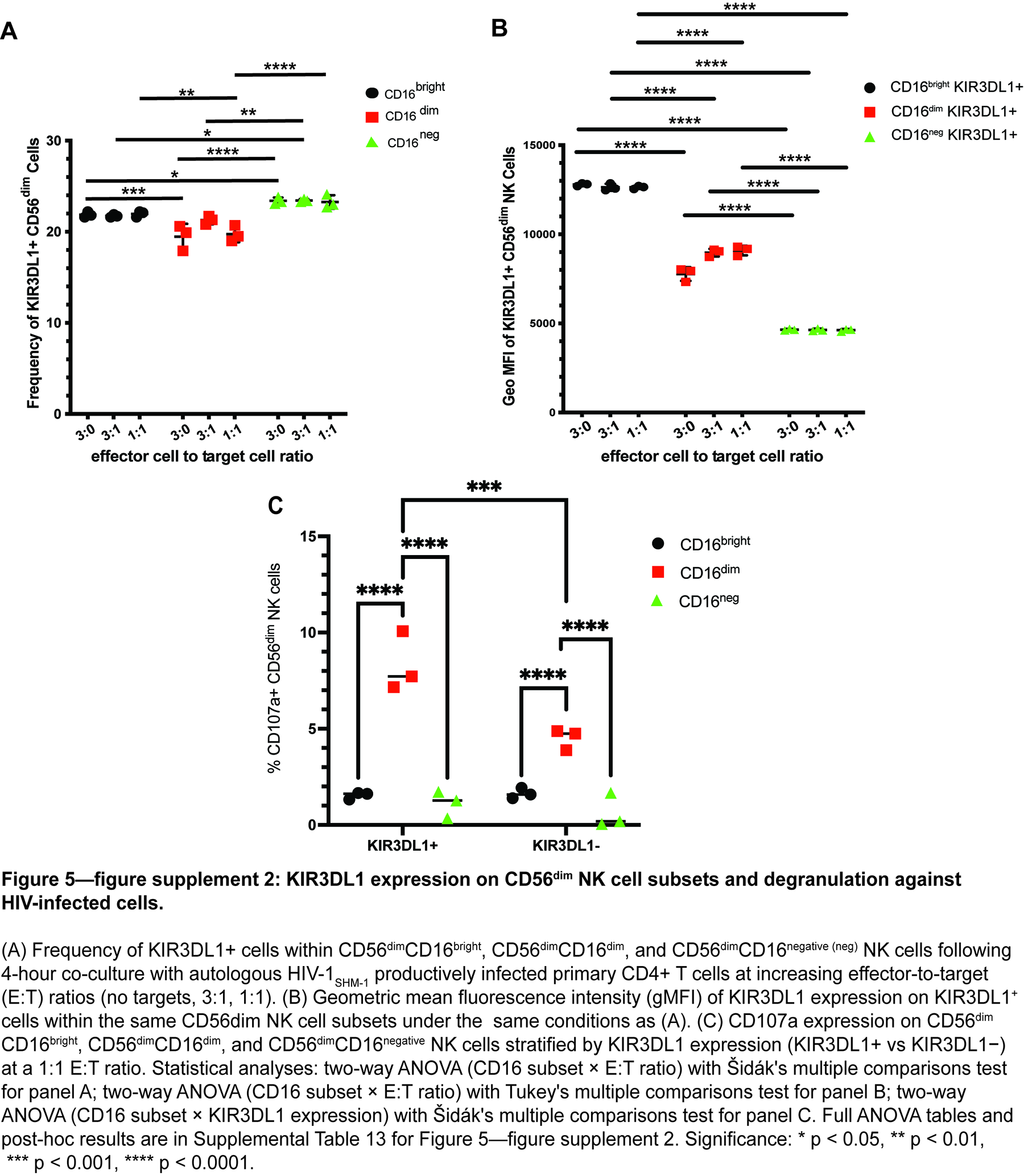

Supplement: Supplement 36 [file media-36.tif]

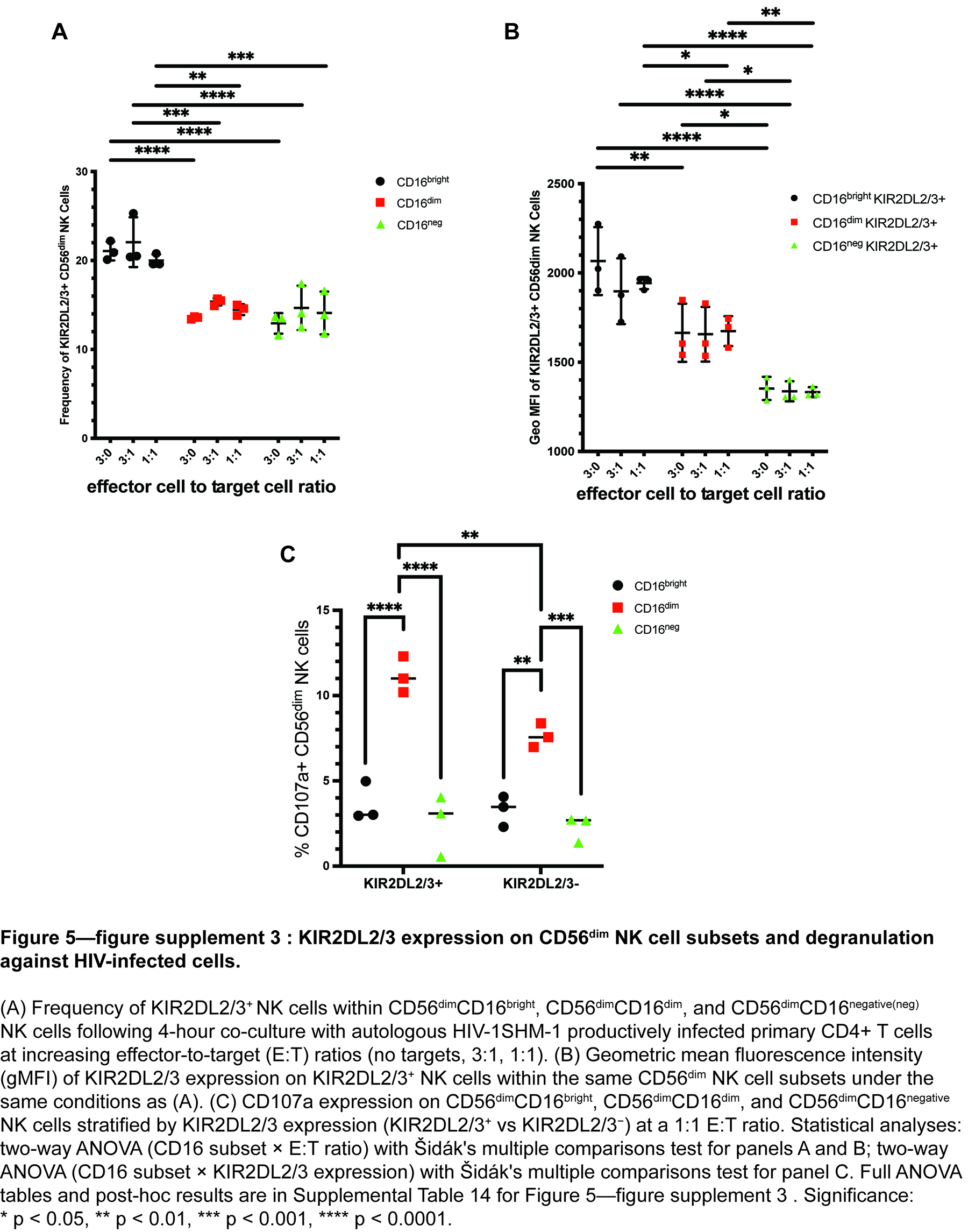

Supplement: Supplement 37 [file media-37.tif]

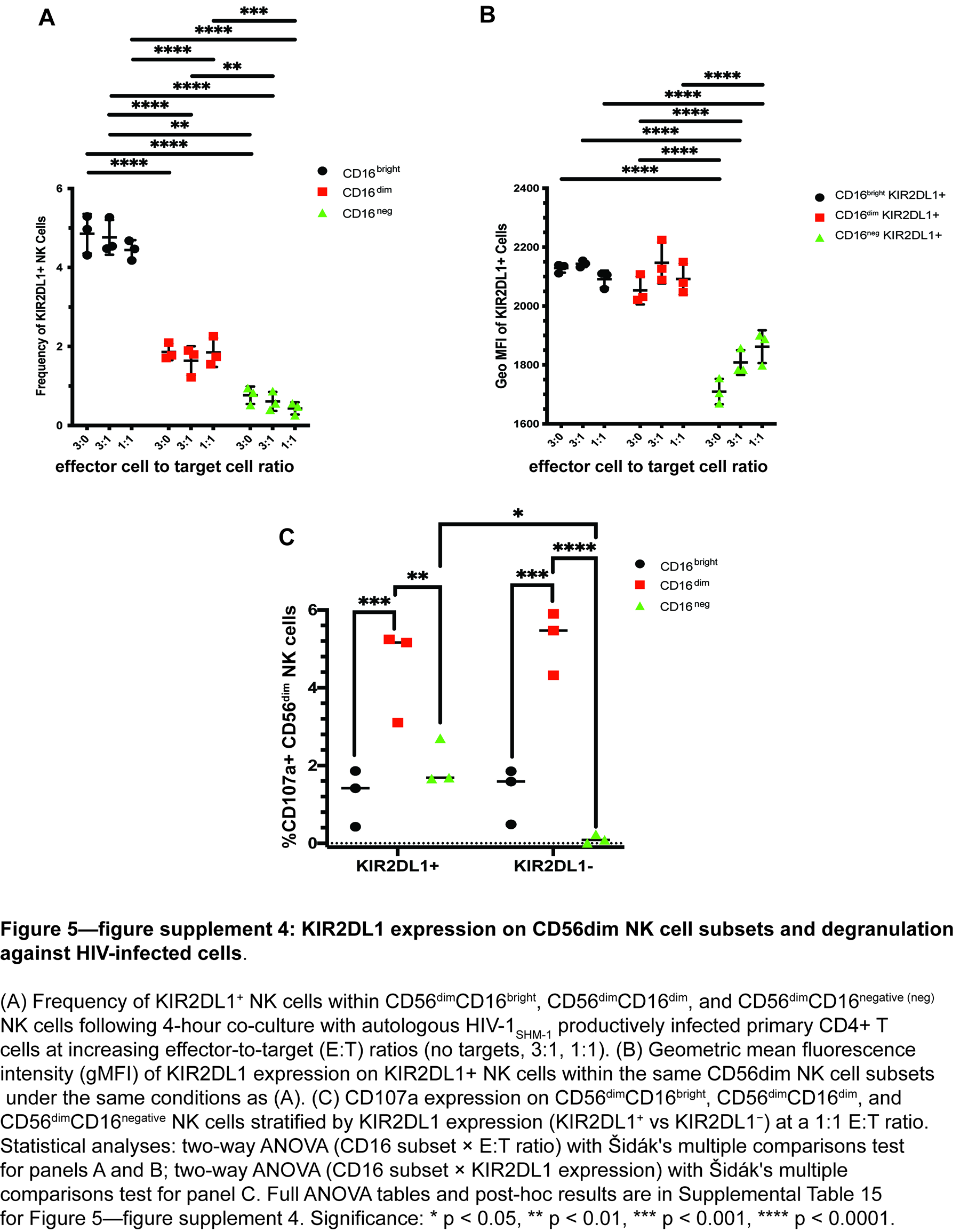

Supplement: Supplement 38 [file media-38.tif]

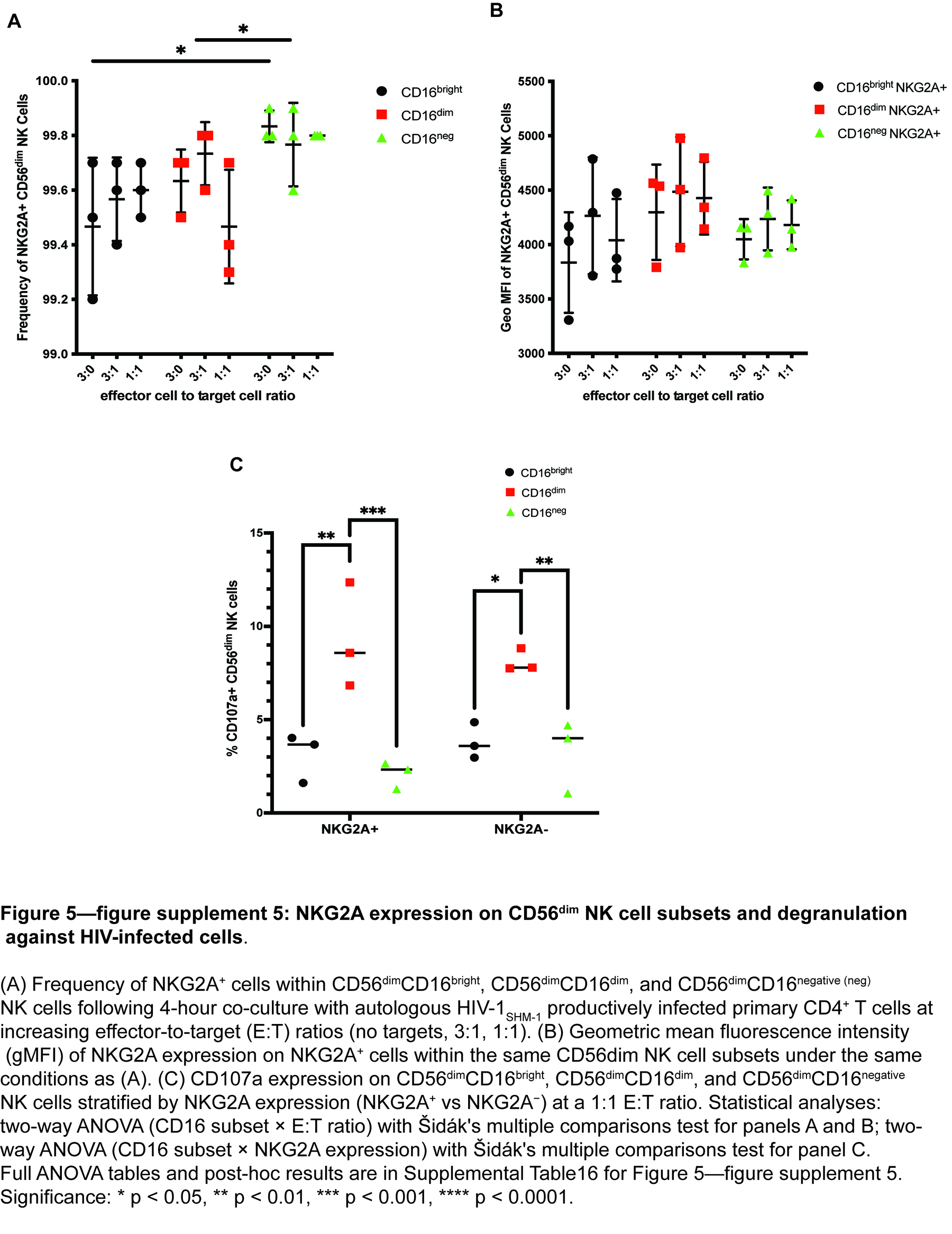

Supplement: Supplement 39 [file media-39.tif]

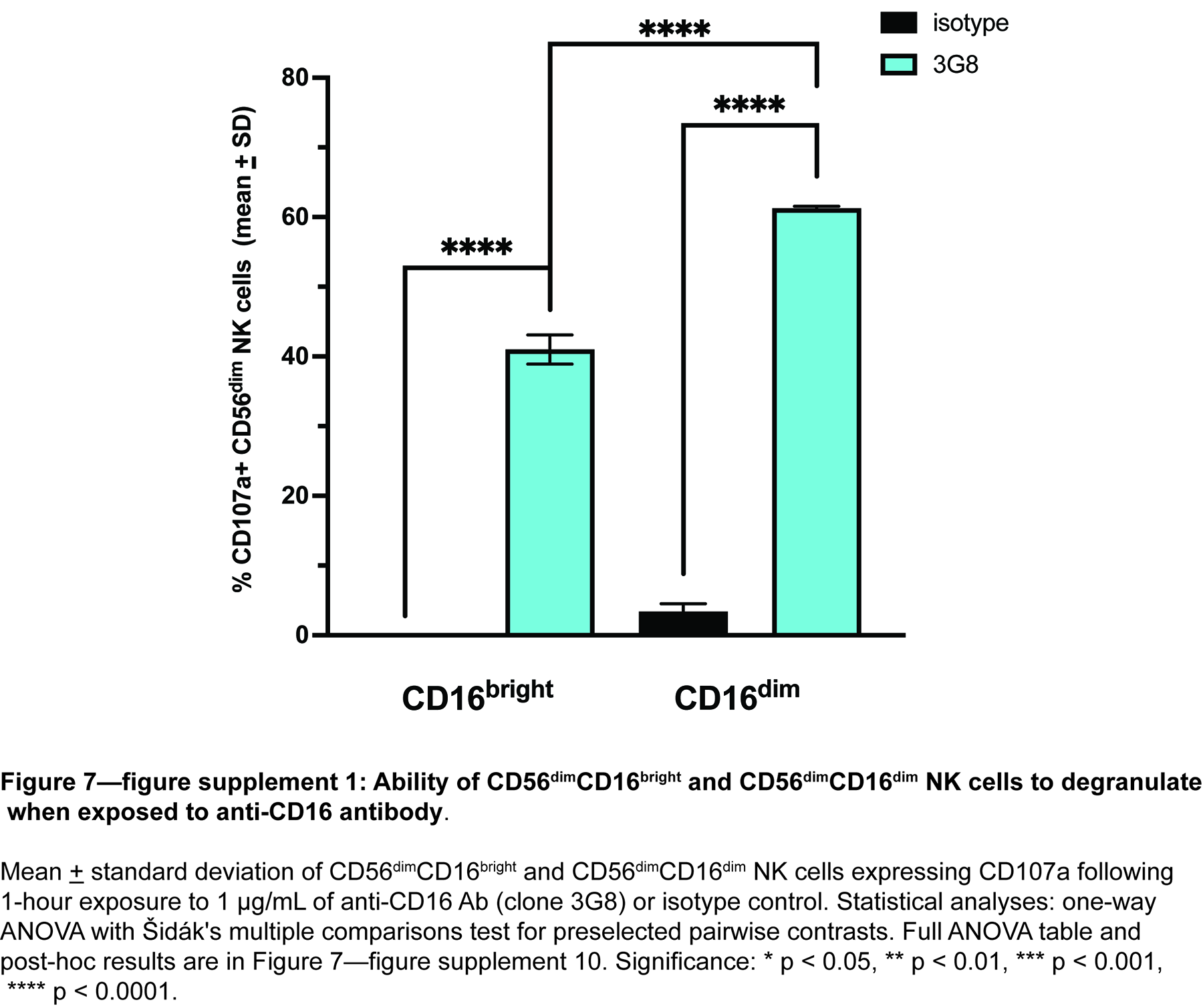

Supplement: Supplement 40 [file media-40.tif]
